# Validation of the Arabic linguistic version of the Overactive Bladder Symptoms Score questionnaire

**DOI:** 10.1080/2090598X.2019.1627061

**Published:** 2019-06-12

**Authors:** M. A. Elbaset, Abdelwahab Hashem, Diaa-Eldin Taha, Mohamad H. Zahran, Ahmed S. El-Hefnawy

**Affiliations:** a Department of Urology, Urology and Nephrology Center, Mansoura University, Mansoura, Egypt; b Department of Urology, Kafr El-Shiekh University, Kafr El-Shiekh, Egypt

**Keywords:** Arabic validation, Overactive Bladder Symptoms Score (OABSS), questionnaire, quality of life (QOL), Over active bladder (OAB), Lower urinary tract symptoms (LUTS)

## Abstract

**Objective**: To validate an Arabic version of the Overactive Bladder Symptom Score (OABSS) questionnaire.

**Patients and methods**: In all, 301 patients were evaluated using the Arabic-translated OABSS. They were divided into four groups: 112 patients with OAB symptoms, 115 healthy individuals with no OAB symptoms, 38 with bladder outlet obstruction (BOO) associated with storage lower urinary tract symptoms (LUTS), and 36 with BOO without storage LUTS. The reliability of the Arabic version was evaluated for internal consistency using Cronbach’s α test. Interdomain associations were examined using Spearman’s correlation coefficient (*r*). The discrimination validity was evaluated using the Mann–Whitney test.

**Results**: Higher internal consistency was found for all OABSS domains in the OAB and BOO groups. There were strong correlations between all domains in the OAB group (*P* < 0.001). Similarly, there were strong correlations between all domains in the BOO group. For discrimination validity, scores were statistically significant higher for all OABSS domains and overall total scores in the OAB and BOO groups compared with their control groups (*P* < 0.001).

**Conclusion**: The Arabic version of OABSS is a reliable and valid instrument that can be used to evaluate symptoms and health-related quality of life in Arabic patients with OAB.

Clinical trial no. (clinicaltrials.gov NCT03533062)

**Abbreviations :** BOO: bladder outlet obstruction; OAB: overactive bladder; OABSS: Overactive Bladder Symptom Score questionnaire; (U)UI: (urgency) urinary incontinence

## Introduction

Overactive bladder (OAB) is a condition characterised by the presence of urinary urgency, frequency and nocturia, with or without urgency urinary incontinence (UUI), in the absence of UTI or other clear pathology [1]. Frequency is the most commonly reported symptom, followed by urgency and UUI, in 85%, 54% and 36%, respectively [2]. OAB is a prevalent, chronic symptom complex that impairs quality of life (QoL). The prevalence amongst the European general population is 11.8% [3].

Treatment objectives are to reduce the occurrence of bothersome symptoms [4]. Several treatments are available for OAB including: bladder and behavioural training, pharmacological treatment, and surgical treatment [5]. Patient self-completed questionnaires are the most suitable method for assessing the patient’s perspective of their LUTS. There are many questionnaires available for use, each fulfilling a different purpose. They may be used directly to assess LUTS for monitoring progress on or after treatment, in epidemiological studies or with economic and health service evaluation. BOO associated with storage LUTS causes embarrassment and disrupts daily life adversely affecting patients’ QoL [6].

The use of a valid objective tool to evaluate OAB symptoms and their effect on QoL became available after the development of the Overactive Bladder Symptom Score (OABSS) questionnaire [7]. This questionnaire was used to objectively compare different treatment groups. Validated versions of the OABSS have been published in the Japanese, Korean, Chinese, and English languages [7–11]. Arabic is one of the most popular languages in the world, being spoken by as many as 420 million people (native and non-native) in the Arab world. It is one of the six official languages of the United Nations.

The present study was conducted to provide a validated version of the OABSS in Arabic. The validation of the Arabic version of this questionnaire will allow its use in future research in all Arabic-speaking countries.

## Patients and methods

The study was approved by the Institutional Review Board. The Arabic linguistic translation of the OABSS was produced using a multi-step process following the guidelines for cross-cultural adaptation of health-related QoL measures. The English version of the OABSS questionnaire was initially translated into Arabic in parallel by two urologists and one independent native Arabic-speaking professional translator who had English as the first foreign language. Simple language was used to be understandable even by individuals with lower education levels. Any differences between the versions were resolved through a consensus meeting between the translator and the urologists. This Arabic version was back-translated by an independent English-speaking professional with Arabic as the first foreign language. Another back-translation was done by a native English-speaking urologist who has Arabic as the first foreign language. The original OABSS and back-translated version were compared and differences were resolved in a second consensus meeting. This revised version (definitive version) was used for the study (Supplementary Appendix).

A pilot test was used to assess whether the questionnaire was clear and appropriate, by interviews with 15 male and 15 female patients who had or did not have OAB symptoms. Interviewed patients described question clarity, simplicity, and comprehensiveness. No difficulties were reported in completing it, so no further changes were made.

The study included four groups; the first group comprised 112 patients with OAB (OAB group). Patients with UTIs, uterine prolapse, mixed UI, chronic pelvic pain syndrome or those who were receiving medications that affected lower urinary tract function, were excluded from the study. The second group (control group) comprised 115 healthy individuals who agreed to complete questionnaire. The third group included 38 newly diagnosed males, diagnosed using the IPSS, DRE, obstructed flow curve and post-void residual urine volume, as having BOO with storage LUTS (BOO group). The fourth group included 36 male patients with BOO without storage LUTS (BOO control group). The Arabic version of the OABSS was completed at baseline and 2 weeks later, with the controls only completing the questionnaires once.

### Sample size calculation

Assuming type I statistical error of 5% and type II statistical error of 5%, we designed our study to have a power of 95%. The expected difference between the study and control group as regard total OABSS was 0.6 [7]. The number of cases suspected to drop out was 10–20%. So, the sample size required to give a significant difference was 90 in each arm.

### Statistical analysis

The reliability of the Arabic OABSS was evaluated for internal consistency using Cronbach’s α for each domain. Domain structures were examined by interdomain associations using Spearman’s correlation coefficient (*r*) [12]. Strong correlation was considered when *r* > 0.5. The discrimination validity was evaluated by comparing the scores of cases with those of controls using the Mann–Whitney test. All statistical tests were done using the IBM Statistical Package for the Social Sciences (SPSS®), version 21 (SPSS Inc., IBM Corp., Armonk, NY, USA); with *P* < 0.05 considered statistically significant.

## Results

Of the 309 patients who were assessed, a total of 301 participants were included in the study. Eight patients were excluded as they did not meet the inclusion criteria. The OAB group included 14 (12.5%) and 98 (87.5%) males and females, respectively; with a mean (SD) age of 37 (14) years. The control group included three (2.6%) males, with a mean (SD) age of 30 (8.1) years. The mean (SD) age in the BOO group and BOO control group were 63 (2.6) and 65 (3.4) years, respectively. In the overall study population, 150 patients (49.8%) had OAB symptoms and 151 (50.2%) did not. In all groups, there were no differences in mean age and gender between the groups.

A high level of internal consistency was observed amongst the four domains answered by all participants. In the OAB groups, there was higher internal consistency for all domains as shown by Cronbach’s α test. There were strong correlations between frequency and nocturia (*r* = 0.68), urgency (*r* = 0.63), and UUI (*r* = 0.67) (*P* < 0.001), and between nocturia and urgency (*r* = 0.52), UUI (*r* = 0.72) (*P* < 0.01), and urgency and UUI (*r* = 0.54) (*P* < 0.001) (Table 1).

**Table 1.** Internal consistency (Cronbach’s α) and interdomain association by Spearman’s correlation coefficient in the OAB group.

Domains	Cronbach’s α	Frequency	Nocturia	Urgency	UUI
		***r (P)***	***r (P)***	***r (P)***	***r (P)***
Frequency	0.73		0.68 (<0.001)	0.63 (<0. 001)	0.67 (<0.001)
Nocturia	0.84	0.68 (<0.001)		0.52 (<0.001)	0.73 (<0.001)
Urgency	0.81	0.63 (<0.001)	0.52 (<0.001)		0.54 (<0.001)
UUI	0.89	0.67 (<0.001)	0.73 (<0.001)	0.54 (< 0.001)	

Likewise, in the BOO group there was high internal consistency for all domains by Cronbach’s test with strong correlations between frequency and nocturia, urgency, and UUI (*P* < 0.001, *P* = 0.003, and *P* < 0.001, respectively), between nocturia and UUI (*P* < 0.001), and between urgency and UUI (*P* < 0.001) (Table 2).

**Table 2.** Internal consistency (Cronbach’s α) and interdomain association by Spearman’s correlation coefficient in the BOO group (BOO with OAB-like symptoms).

Domains	Cronbach’s α	Frequency	Nocturia	Urgency	UUI
		***r (P)***	***r (P)***	***r (P)***	***r (P)***
Frequency	0.82		0.69 (<0.001)	0.50 (0.003)	0.62 (<0.001)
Nocturia	0.77	0.69 (<0.001)		0.34 (0.01)	0.55 (<0.001)
Urgency	0.92	0.50 (0.003)	0.34 (0.01)		0.68 (<0.001)
UUI	0.87	0.62 (<0.001)	0.55 (<0.001)	0.68 (< 0.001)	

Discriminant validity was assessed by comparing OABSS between patients with OAB and control groups, which revealed statistically significantly higher scores in all domains and total scores in the OAB group (*P* < 0.001). The same results were noted when comparing the BOO group with its control group (Tables 3 and 4).

**Table 3.** The discrimination properties of the Arabic OABSS between patients with OAB and controls.

OABSS, median (range)	Patients (*n* = 112)	Controls (*n* = 115)	P*
Frequency	1 (0–2)	0 (0–1)	<0.001
Nocturia	3 (0–3)	1 (0–3)	<0.001
Urgency	4 (2–5)	0 (0–3)	<0.001
UUI	4 (2–5)	0 (0–1)	<0.001
Total OABSS	11 (8–15)	2 (0–4)	<0.001

*Mann–Whitney test.

**Table 4.** The discrimination properties of the Arabic OABSS between patients with BOO with OAB-like symptoms (BOO group) and controls.

OABSS, median (range)	Patients (*n* = 38)	Controls (*n* = 38)	P*
Frequency	1 (0–2)	0 (0–2)	<0.001
Nocturia	3 (2–3)	2 (0–3)	<0.001
Urgency	4 (2–5)	1 (0–4)	<0.001
UUI	2.5 (3–5)	1 (0–2)	<0.001
Total OABSS	12 (7–15)	5 (1–7)	<0.001

*Mann–Whitney test.

## Discussion

Questionnaires and symptom tools are used by clinicians to evaluate patient’s symptoms and help develop treatment strategies. The limited availability of trained commentators is problematic for clinicians and patients in terms of evaluation and treatment. The OABSS is a four-item questionnaire that quantifies OAB symptoms in a single score. The OABSS was originally developed by bilingual Japanese urologists, concurrently in both Japanese and English. The score is the simple sum of four symptom scores, which address daytime voiding, night-time voiding, urgency, and UUI. OAB was diagnosed based on the last ICS standardisation of terminology, which defined the condition by the presence of frequency, nocturia, urgency and sometimes UUI, in the absence of specific pathology or previous irradiation. It is noteworthy that a significant percentage of patients with OAB may not have detrusor overactivity on urodynamics. In the present study, urodynamic studies were carried out whenever indicated, e.g. in patients with a poor response to medical treatment. The appropriateness of the translated English version of the OABSS was confirmed after assessing translation quality and carrying out cognitive debriefing [13]. BOO, caused by BPH, is a health problem distressing >50% of men aged >40 years. Pathogenesis of BPH is thought not only to involve obstruction, but is also associated with bladder dysfunction and storage LUTS (OAB-like symptoms). Commonly, OAB-like symptoms associated with BPH include not only voiding symptoms, but also storage symptoms (e.g. frequency, nocturia, and UI), which are humiliating and disruptive to daily life. Concerning the interaction between BOO and OAB, it has been reported that 47% of patients with BOO have OAB-like symptoms, similarly it has been reported that the degree of bother in older men correlated with the degree of OAB symptoms in patients with BOO and OAB at baseline and after treatment [6].

The reliability of the Arabic version was confirmed by the high and intermediate internal consistency and good interdomain correlations in the OAB and BOO groups (Tables 1 and 2), which is in agreement with other languages in which the OABSS was translated, e.g. Korean, Chinese, English and Persian, dealing with OAB with or without treatment [14,15]. The discrimination validity was obvious by the significant difference in all domain scores between patients with OAB and BOO associated storage LUTS and healthy individuals (Tables 3 and 4). This agreement can be attributed to the high discriminatory power and good psychometric properties of the original English-validated questionnaire and using a multi-step approach in the translation process, which consisted of forward and backward translations, and this method is now considered the standard for linguistic validation of patient-reported outcomes.

Consequently the results of the present study show that the Arabic language version of the OABSS can be used by urologists to easily and quickly assess patients without the aid of interpreter services. It is imperative that clinicians not only understand the prevalence of OAB in the community but also, its growing trend in elderly being under reported as it is considered a normal result of ageing progression. Screening of patients with the OABSS will help to elucidate and grade OAB symptoms in both young and geriatric populations.

The present study was conducted in a tertiary specialised urology clinic and that the content of each question was evaluated by both the subjects and the expert panel adds strength to the study, in addition to wider based population in both males and females with different associated diseases. Limitations of the present study are that it was conducted in a single centre. Data were not collected in different Arabic speaking countries, so it is possible that there are different interpretations to questions based on the Arabic tongue variation. We did not compare the Arabic version of OABSS with other health-related QoL scores, this is attributed to the fact that there is no defined Arabic translated questionnaire for OAB, as well as OAB not only had a negative impact on urinary symptoms, but also on other health-related QoL, such as general health. Another reason that QoL questionnaires were not compared with the Arabic version of the OABSS was because a considerable proportion of our patients were not educated or had low levels of education that could affect their ability to answer such questionnaires. Another limitation of the present study was an inability to assess test–retest reliability, as we mentioned due to the low educational level in many patients. Also, we did not include older aged females with OAB for assessment.

## Conclusion

The Arabic version of the OABSS questionnaire is a valuable self-administered assessment tool, which allows assessment of OAB symptoms and patient’s subjective perceptions in both males and females in Arabic-speaking populations.

Clinical trial no. (clinicaltrials.gov NCT03533062)

## Supplemental material

Supplemental Appendix_Elbaset et al.
