# Supplementary material for: Validation of the Arabic linguistic version of the Overactive Bladder Symptoms Score questionnaire
Source: Arab J Urol. 2019 Jun 12;17(4):265–9. doi: 10.1080/2090598X.2019.1627061 (PMC6830271; doi:10.1080/2090598X.2019.1627061)
Supplement: Supplemental Appendix_Elbaset et al. [file TAJU_A_1627061_SM2613.docx]

**Supplementary Appendix**

| 0 | اقل من 7 مرات | عدد مرات دخول الحمام بالنهار  (منذ الإستيقاظ من النوم صباحا وحتى النوم ليلا) |
| --- | --- | --- |
| 1 | 8- 14 |  |
| 2 | اكثر من 15 |  |
| 0 | 0 | عدد مرات دخول الحمام بالليل  (منذ الذهاب للنوم مساء وحتى الإستيقاظ فى الصباح) |
| 1 | مره واحده |  |
| 2 | مرتان |  |
| 3 | اكثر من 3مرات |  |
| 0 | مطلقا | الالحاح البولي  (احساس مفاجىء بالتبول ولا يمكن تأجيله) |
| 1 | اقل من مره فى الإسبوع |  |
| 2 | مره او اكثر فى الإسبوع |  |
| 3 | مره فى الإسبوع |  |
| 4 | 2-4 مرات فى الإسبوع |  |
| 5 | 5 مرات فى اليوم او اكثر |  |
| 0 | مطلقا | السلس البولي  (احساس مفاجىء بالتبول ولا يمكن تأجيله وقد يؤدى الى سقوط البول لا اراديا) |
| 1 | اقل من مره فى الإسبوع |  |
| 2 | مره او اكثر فى الإسبوع |  |
| 3 | مره فى الإسبوع |  |
| 4 | 2-4 مرات فى الإسبوع |  |
| 5 | 5 مرات فى اليوم او اكثر |  |

**Arabic-validated version of the OABSS.**

| Question | Frequency | Score |
| --- | --- | --- |
| Daytime frequency  How many times do you typically urinate from waking in the morning until sleeping at night? | ≤7 | 0 |
|  | 8–14 | 1 |
|  | ≥15 | 2 |
| Night-time frequency  How many times do you typically urinate from sleeping at night until waking in the morning? | 0 | 0 |
|  | 1 | 1 |
|  | 2 | 2 |
|  | ≥3 | 3 |
| Urgency  How often do you have a sudden desire to urinate, which is difficult to defer? | Not at all | 0 |
|  | Less than once a week | 1 |
|  | Once a week or more | 2 |
|  | About once a week | 3 |
|  | 2–4-times a day | 4 |
|  | 5-times a day or more | 5 |
| Urgency Incontinence  How often do you leak urine because you cannot defer the sudden desire to urinate? | Not at all | 0 |
|  | Less than once a week | 1 |
|  | Once a week or more | 2 |
|  | About once a week | 3 |
|  | 2–4-times a day | 4 |
|  | 5-times a day or more | 5 |

**English version of the OABSS.**
